# The role of alternative energy and globalization in decarbonization prospects of the oil-producing African economies

**DOI:** 10.1007/s11356-023-26581-6

**Published:** 2023-03-28

**Authors:** Stephen Taiwo Onifade, Savaş Erdoğan, Andrew Adewale Alola

**Affiliations:** 1 Faculty of Economics and Administrative Sciences, KTO Karatay University, Konya, Turkey; 2 Department of Economics, Şelcuk University, Konya, Turkey; 3 CREDS-Centre for Research On Digitalization and Sustainability, Inland Norway University of Applied Sciences, Elverum, 2418 Norway; 4 Faculty of Economics, Administrative and Social Sciences, Nisantasi University, Istanbul, Turkey

**Keywords:** Africa, Renewable energy, Fossil energy, Greenhouse gas emission, Sustainable development

## Abstract

This study assesses the environmental impacts of the energy mix of mainly oil-producing African nations. The economic aspects of decarbonization prospects were also viewed from the perspectives of fossil energy dependence among the countries. More insights on the impacts of energy mix on decarbonization prospects were also provided on a country-specific analysis basis via the application of second-generation econometric techniques in assessing carbon emission levels across the countries between 1990 and 2015. From the results, only renewable resources proved to be a significant decarbonization tool among the understudied oil-rich economies. Moreover, the consequences of the trio of fossil fuel consumption, income growth, and globalization are diametrically opposed to achieving decarbonization as the rise in their usage significantly acts as pollutant-inducing tools. The validity of the environmental Kuznets curve (EKC) conjecture was also upheld for the combined analysis of the panel countries. The study thus opined that the reduction in conventional energy dependence will enhance environmental quality. Consequently, given the advantages of the geographical locations of these countries in Africa, concerted strategies for more investment in clean renewable energy sources like solar and wind were suggested to policymakers among other recommendations.

## Introduction

Human activities have continued to trigger environmental degradation across the globe (Cop et al. 2020; Ike et al. 2020; Umar et al. 2021). At the same time, the world is witnessing a growing need for sustainable and reliable energy sources at affordable costs, and the need for collective actions to preserve the global environment is also on the rise. As such, the 7th and 13th goals of the seventeen (17) sustainable development goals (SDGs) of the United Nations are set towards achieving energy for sustainable development and necessary climate actions. Until now, a huge chunk of the global energy demand is being met by fossil energy resources like oil, gas, and coal (Alola and Onifade 2022; Gyamfi et al. 2022). Although these fossil energy resource endowments are largely unevenly distributed across the globe, the rising trends in globalization have however fostered the rates of energy interdependency through trades among countries thereby stimulating global energy consumption. This development helps nations to overcome their domestic energy supply deficits. However, meeting the global energy demand through fossil fuels also comes at a cost to the environment since it leads to greenhouse gas (GHG) emissions (IPCC 2019; Gyamfi and Adebayo 2022). Hence, various stakeholders have been engaged in many international submits to support greenhouse emission reduction targets right from the Kyoto Protocol of 1997 until the Paris Agreement of 2015. Since then, emission reduction goals and their scopes have continued to expand and dominate the center of discussion in major climate submits as seen in the most recent 27th United Nations Climate Change Conference (COP27) in Egypt (Hussain and Mahase 2022).

**Fig. 1:**
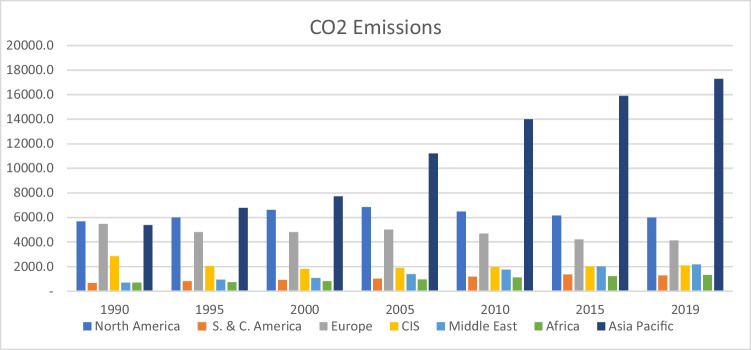
Growth of global CO_2_ emissions by regions. Source: Authors’ computations using Statistical Review of World Energy (BP 2020). CIS denotes the Commonwealth of Independent States, while S. and C. are South and Central America. Data is given in million tons of carbon dioxide

**Fig. 2:**
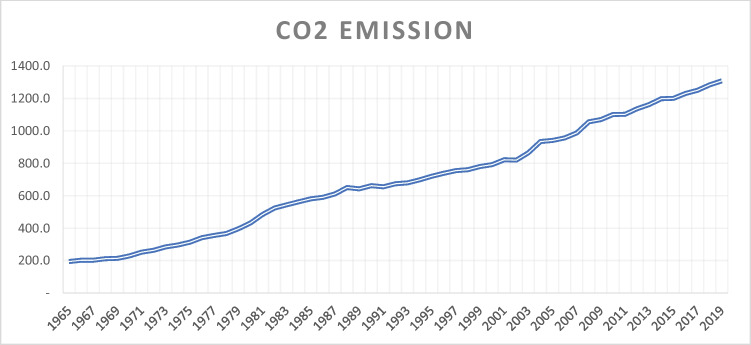
Trend of CO_2_ emission in Africa (1965–2019). Source: Authors’ computations using data from BP (2020). Data is given in million tons of carbon dioxide

Based on available data (BP 2020), the countries in Africa and those in the South and Central America region contribute the least amount to global CO_2_ emission as of 2019 as shown in Fig. 1. Although Africa currently contributes a significantly low proportion of the global CO_2_ emission, however, given the ongoing era of globalization, the trend of CO_2_ emission in Africa between 1965 and 2019 reflects a significant rise as shown in Fig. 2. For instance, the amount of CO_2_ emitted in 2019 is put at 1308.52 (million tons) compared to the estimated 193.90 (million tons) of emissions in the mid-1960s (BP 2020) which represents an increase of about 575% growth in emissions over the period. Thus, as the continent continues to experience growing energy demands over time, the continuation on the path of the current energy mix among many African nations may imply more setbacks for the global greenhouse gas (GHG) emission reduction targets. Moreover, the economic stability of many African states and their inherent integration into the global economy is largely dependent on fossil resource endowments (Onifade 2022). Therefore, there is a need to better understand the role of alternative energy sources and globalization in the decarbonization prospects of the oil-producing African economies.

Trade in oil and gas has been the backbone of some notable oil-exporting countries in Africa, and more countries are joining the league as new oil and gas fields are being discovered on the continent. It has been observed that there is also a high possibility of discovering more oil and gas reserves in Africa since exploration activities are relatively low in previous decades (Graham and Ovadia 2019). Also, the oil and gas exporters in Africa have earned over $1.7 trillion from trade in oil and gas and these earnings account for about 25% of the GDP growth in Africa in the 2000s (International Energy Agency 2019). This huge revenue has made many oil-exporting African countries reliant on their oil industry but without experiencing a high level of poverty and inequality, political instability, lack of basic amenities, mismanagement and corruption, and unemployment (Abid and Sekrafi 2020; Botha 2008; Asongu et al. 2020; Çevik et al. 2020; Hakan et al. 2022). Moreover, the woes of the continent are now being further exacerbated by environmental degradation challenges in recent years. Hence, taking into cognizance the crucial risks posed by climate change in Africa in our increasingly globalized world, this research seeks to:

i. Explore the environmental impacts of the prevailing energy dynamics of mainly oil-producing nations amidst examining the validity of the EKC hypothesis.
ii. Explore the environmental consequences of globalization within the context of oil-producing African states.
iii. Review recent economic challenges in oil-exporting African states in our increasingly globalized world.

In addition, the study significantly takes care of some empirical limitations like the issues of cross-sectional dependence through the adopted techniques thereby avoiding notable flaws in extant studies. Moving on from the introduction, the rest of the study features “Literature review” and “Empirical methods” which encapsulate the literature review and empirical methods accordingly. The fourth section encapsulates the results discussions, while the fifth section raps up the study with the conclusion and policy suggestions thereafter.

## Literature review

### Energy resources trade of the oil-exporting African states in a globalized world

Trade in oil and gas is crucial to the economic stability of many oil-exporting African countries (Taiwo et al. 2020). In 2019, the total global crude oil export based on production was estimated at 45.18 million barrels per day (mb/day) (Organization of Petroleum Exporting Countries 2020) while the country was the third-largest exporter at about 14.24% (Organization of Petroleum Exporting Countries 2020). Over the years, several African countries have been able to attract large foreign direct investment (FDI) in their oil and gas sector from various giant multinational oil corporations partly due to the presence of these resources in commercial quantity as shown in Tables 1 and 2. Overall, it can be deduced that these countries have enjoyed the benefits of globalization as it is often seen as a major tool for fostering economic integration and redistribution of scarce resources across international boundaries to meet certain human needs including energy demands among others. Besides, it has been observed that the trends of globalization reveal how the world economies are interdependent and interconnected especially through trade and foreign direct investment which cut across both advanced and emerging economies (Balcilar et al. 2023; Yussif et al. 2022; Adebayo et al. 2022; Khatir et al. 2022; Dingru et al. 2023). We utilized the KOF Globalization index of the KOF Swiss Economic Institute in this research. This index measures globalization from three major perspectives including economic perspectives, political perspectives, and social perspectives. From these perspectives, the index publishes various dimensions of globalization including trade globalization, financial globalization, interpersonal globalization, cultural globalization, information globalization, and political globalization. The index was originally initiated by German economist Dreher (2006). This index has been widely utilized, revised, and applied in globalization-related studies in various fields (Gygli et al. 2019; Liu et al. 2020).

**Table 1:** Africa’s proved oil reserves (billion barrels) 2010–2019

Country	2010	2011	2012	2013	2014	2015	2016	2017	2018	2019	Share 1	Share 2
Algeria	12.2	12.2	12.2	12.2	12.2	12.2	12.2	12.2	12.2	12.2	0.7%	9.71%
Angola	9.1	9.1	9.1	9.0	8.4	9.5	9.5	8.4	8.2	8.2	0.5%	6.49%
Chad	1.5	1.5	1.5	1.5	1.5	1.5	1.5	1.5	1.5	1.5	0.1%	1.19%
Rep of Congo*	2.0	2.0	2.0	2.7	2.9	3.0	3.0	3.0	3.0	3.0	0.2%	2.37%
Egypt	4.5	4.3	4.2	3.9	3.7	3.5	3.4	3.3	3.1	3.1	0.2%	2.45%
Equatorial G*	1.7	1.7	1.7	1.7	1.1	1.1	1.1	1.1	1.1	1.1	0.1%	0.88%
Gabon	2.0	2.0	2.0	2.0	2.0	2.0	2.0	2.0	2.0	2.0	0.1%	1.59%
Libya	47.1	48.0	48.5	48.4	48.4	48.4	48.4	48.4	48.4	48.4	2.8%	38.48%
Nigeria	37.2	36.2	37.1	37.1	37.4	37.1	37.5	37.5	37.0	37.0	2.1%	29.41%
South Sudan	n/a	n/a	3.5	3.5	3.5	3.5	3.5	3.5	3.5	3.5	0.2%	2.78%
Sudan	5.0	5.0	1.5	1.5	1.5	1.5	1.5	1.5	1.5	1.5	0.1%	1.19%
Tunisia	0.4	0.4	0.4	0.4	0.4	0.4	0.4	0.4	0.4	0.4	0.025%	0.34%
Other Africa	2.3	2.2	3.7	3.7	3.7	4.0	4.0	3.9	3.9	3.9	0.2%	3.12%
Total Africa	**124.9**	**124.6**	**127.4**	**127.5**	**126.8**	**127.6**	**127.9**	**126.7**	**125.7**	**125.7**	**7.2%**	**100%**

Source: Author’s compilation using data from British Petroleum (2020). Share 1 is the share of Africa in the global proved oil reserves, while Share 2 represents the share in total Africa’s proved oil reserves

*Equatorial Guinea, and the Republic of Congo

**Table 2:** Africa’s proved gas reserves (trillion cubic meters) from the end of 1999 to the end of 2019

Country	At end 1999	At end 2009	At end 2018	At end 2019	Share 1	Share 2
Algeria	4.3505	4.3351	4.3351	4.3351	2.2%	29.04993
Egypt	1.177138	2.107875	2.137713	2.137713	1.1%	14.32502
Libya	1.24925	1.47155	1.429654	1.429654	0.7%	9.580255
Nigeria	3.3364	5.0274	5.391247	5.391247	2.7%	36.12728
Other Africa	0.844453	1.230074	1.370894	1.629212	0.8%	10.91751
Total Africa	10.95774	14.172	14.66461	14.92293	7.5%	100%

Source: Author’s compilation using data from British Petroleum (2020). Share 1 is the share of Africa in global proved natural gas reserves, while Share 2 represents the share in total Africa’s proved gas reserves

### Theoretical and empirical literature

One of the major theories that have received attention among researchers when linking energy use to environmental sustainability and decarbonization prospects among countries is the environmental Kuznets curve (EKC) hypothesis. This theory provides the basic influencing mechanism among energy indicators and environmental performances. As enunciated in the early works of Kuznets (1955), the EKC hypothesis posits an inverted U-shape interaction amidst environmental deterioration from pollutant elements and GDP growth indicators on the premise that rapid economic expansion would pave way for an initial rise in the level of degradation of the environment, but the effect of which will later be counterbalanced as growth continues to expand over time. However, available empirical evidence on the validity of this theoretical underpinning remains largely mixed among extant studies.

Currently, there is a growing number of empirical studies addressing the challenges of CO_2_ emission and how to foster decarbonization in the literature. A review of the literature on energy use, GDP growth, globalization, and CO_2_ emission relationship divulges a growing number of empirical studies. However, the largest proportion has concentrated on addressing this issue in developed economies like the United States (US), the United Kingdom (UK), and other bodies of economic integration like the European Union and the Organization for Economic Cooperation and Development (Adedoyin and Zakari 2020, Bekun et al. 2019; González et al. 2014; Khan et al. 2021). The case of other blocs such as the Organization of Petroleum Exporting Countries and China as the leading carbon-emitting country has also received substantial attention in the literature (Alola et al. 2021; Bekun et al. 2021; Ilham et al. 2021; Onifade et al. 2021; Shahbaz et al. 2022; Zhao et al. 2022). Many of the studies have attempted to explore the factors contributing to emission levels, and the outcomes vary from one study to another, especially in the context of the indicators used, the methods, and in terms of the magnitudes of the impacts of the adopted indicators on the emission level.

Bekun et al. (2019) considered the energy-emission link in the case of 16 EU countries using the PMG-ARDL technique. The study also includes the impact of economic growth. They discovered that carbon emission is increased by both fossil energy consumption and economic growth in the EU. However, their study does not explore whether globalization has an important role in the understudied framework. Besides, the study also bypasses the possibility of exploring the EKC for the 16 EU nations. However, Le and Ozturk (2020) considered the carbon emission impacts of both energy use and globalization among 47 emerging markets and developing economies. They also considered the roles of economic growth in the study. By the latter step, they were able to draw a conclusion on the validity of the EKC. From the results of the study, they noted that carbon emission levels are increased by both globalization and energy use. They also further pointed out that the EKC hypothesis is valid among these emerging economies. Many other studies have also come up with evidence for or against the EKC validity (Shahbaz et al. 2016; Xu et al. 2022; Mahmood et al. 2019; Xie et al. 2022).

On the other hand, attention is gradually starting to rise on the subject matter for the case of African countries. However, this attempt has only been well-documented in a few studies (Ben Jebli et al. 2015; Iorember et al. 2020, 2021; Nwani et al. 2021). Ben Jebli et al. (2015) explored the causality links between growth, renewable energy consumption, and carbon emissions in twenty-four sub-Saharan African nations. Their results show varying degrees of causal nexuses among these variables, and they also noted that the EKC is not valid in Sub-Saharan Africa. However, their findings are at variance with the results of the study of Iorember et al. (2020) which reveals that the EKC is valid. It is however crucial to note that the scope of the latter study is smaller compared to Ben Jebli et al. (2015) since they only examined the specific case of the Nigerian economy among other sub-Saharan African states. Other studies on Africa like Nwani et al. (2021) for the case of the Algerian economy have also bypassed the examination of the EKC. Therefore, the growing debate on the validity of the hypothesis is still open to further investigation.

Overall, despite the growing number of empirical studies addressing the challenges of global CO_2_ emission and how to foster decarbonization in the literature, most studies have disproportionally concentrated on addressing this issue in developed economies while some have also ignored the potential roles of globalization in the dynamics. Hence, taking into cognizance the crucial risks posed by climate change in Africa in our increasingly globalized world, this study specifically explores the role of alternative energy and globalization in the decarbonization prospects of mainly oil-producing African economies.

## Empirical methods

To examine the impacts of fossil energy use and renewable energy sources in an attempt to explore the significance of energy mix for both environmental and economic sustainability of African oil-exporting countries, we proposed the energy mix indicator model for impact analysis on carbon emission while capturing the influence of globalization and income levels among the countries, as framed in Eq. 1.

1 $$\mathrm{Ln}{{\mathrm{CO}}_{2}}_{it}={\alpha }_{0}+{\alpha }_{1}{\mathrm{LnPY}}_{it}+{\alpha }_{2}\mathrm{Ln}{\mathrm{PY}2}_{it}+{\alpha }_{3}{\mathrm{LnFFE}}_{it}+{\alpha }_{2}{\mathrm{LnRWE}}_{it}+{\alpha }_{6}{\mathrm{LnATE}}_{it}+{\alpha }_{7}{\mathrm{LnGZ}}_{it}+{\varepsilon }_{it}$$

Full details about the variables presented in Eq. 1 are given in Table 3. Due to the lack of data availability, the scope of the present study covers the period (1990–2015) for nine (9) countries including Algeria, Nigeria, Angola, Egypt, the Republic of Congo, Gabon, Sudan, Tunisia, and South Africa. Although SA is not a notable oil-exporter, however, it is among the leading carbon-intensive nations in the world with a significant proportion of its electricity generation coming from coal which is another prominent fossil energy source (Eberhard 2011).

**Table 3:** Data information

Symbols	Variables	Sources
CO_2_	The amount of carbon dioxide emissions in metric tons per capita	World Bank database
PY	Real GDP per capita evaluated in value of current US$	World Bank database
FFE	Fossil fuel energy consumption obtained as a % of the total energy use	World Bank database
RWE	Renewable energy consumption is taken as a % of the total final energy consumption for the countries	World Bank database
ATE	Alternative and nuclear energy are given as a % of the total energy consumption	World Bank database
GZ	Globalization KOF globalization index	KOF index (2020)

Sources: The author’s compilation using data from the World Bank Database and the globalization index of the KOF Swiss Economic Institute (Gygli et al. 2019)

### Estimation procedures

This study utilizes a combination of second-generation panel data analytical approaches. The adoption of the methodologies was inspired by the crucial need to accommodate the statistical features of the obtained panel data based on the outcomes of necessary pre-estimation tests. Firstly, we presented the simple statistics that describe the analyzed data in Table 4. The correlation statistics in Table 4 show a weak positive correlation between income levels and carbon emission levels but a strong positive correlation with fossil fuel use and globalization. Both renewables and alternatives are negatively correlated with emission levels.

**Table 4:** Summary statistics

Variables	LnCO_2_	LnPY	LnPY^2^	LnFFE	LnRWE	LnATE	LnGZ
Mean	0.1338	1.6662	4.1901	1.6505	1.3394	−0.1300	1.6937
Median	0.2513	1.6351	2.6738	1.5920	1.7848	0.1220	1.6935
Maximum	0.9991	10.146	102.94	1.9999	1.9485	0.6400	1.8499
Minimum	−0.9717	−0.0008	6.95E −0	1.0459	−1.2294	−1.9000	1.4663
Std. Dev	0.5023	1.1915	11.29745	0.3008	0.7592	0.6397	0.0908
Observations	234	234	234	234	234	234	234
Correlation matrix							
LnCO_2_	1						
* p*-value	----						
LnPY	0.1119c	1					
* p*-value	(0.0874)	-----					
LnPY^2^	−0.0868	0.9357a	1				
* p*-value	(0.1857)	(0.0000)	-----				
LnFFE	0.7066a	0.0536	−0.0834	1			
* p*-value	(0.0000)	(0.4137)	(0.2035)	-----			
LnRWE	−0.4911a	−0.0601	0.0610	−0.7743a	1		
* p*-value	(0.0000)	(0.3600)	(0.3527)	(0.0000)	-----		
LnATE	−0.0103	−0.0399	−0.0044	−0.2516a	0.6114a	1	
* p*-value	(0.8754)	(0.5431)	(0.9455)	(0.0001)	(0.0000)	-----	
LnGZ	0.6216a	−0.1141c	−0.2451a	0.6125a	−0.3423a	−0.0703	1
* p*-value	(0.0000)	(0.0814)	(0.0002)	(0.0000)	(0.0000)	(0.2837)	-----

a and c signify statistical significance of estimates at 1% and 10% levels accordingly

Given this brief information about the variables, next, we proceed to check the panel unit root properties, but before then, a cross-sectional dependency (CD) test was examined. We opted for the CD test considering that the panel-data models for the oil-exporting countries in this research are most likely to be cross-sectionally dependent especially as economic activities are likely bound to be closely linked such that the error component of the study is influenced by related common shocks among the countries. The significance of the CD test has been spelled out in some fundamental works (Chudik et al. 2016; Pesaran 2007).

2 $${Y}_{it}= {\delta }_{i}+{\alpha }_{i}{X}_{it}+{\mu }_{it}$$

3 $$LM=T{\textstyle\;\sum\nolimits_{i=1}^{N-1}\sum\nolimits_{J=i+1}^N\;{\mathrm\rho^\wedge}_{ij}^{2}}\,{\chi_N^2}_{(N-1)/2}$$

Given the generalized panel relationship model in Eq. 2 where the cross-section dimension (*i*) ranges from 1 to *N* and the time period (*t*) from 1 to *T*, the null hypothesis of the absence of cross-section dependence shows that Cov(${\mu }_{it}$,${\mu }_{jt}$) = 0, while the alternative argues for the presence of CD in at least a pair of the cross-sections such that Cov(${\mu }_{it}$,${\mu }_{jt}$) ≠ 0. Following the OLS estimation of Eq. (2), we reported the outputs from the Lagrange Multiplier (LM) approach of Breusch and Pagan (1980) where the pair-wise correlation of the obtained residuals is denoted by *ρˆ*_*ij*_.

4 $$CD=\sqrt{\left(\frac{2T}{N(N-1)}\right)}\left(\sum\nolimits_{i=1}^{N-1}\sum\nolimits_{J=i+1}^N {\rho^{\wedge}}_{ij} \right)$$

5 $$\mathrm{\rho^\wedge}_{ji}={\mathrm\rho^\wedge}_{ij}=\frac{\sum\nolimits_{t-1}^T\;\mathrm\mu^{\wedge}_{\;i,t}\;\mathrm\mu^{\wedge}_{\;j,t}}{\left(\sum_{t=1}^T\;\mathrm\mu^{\wedge 2}_{\;it}\right)^\frac12\left(\sum\nolimits_{t=1}^T\;\mathrm\mu^{\wedge 2}_{jt}\right)^\frac12}$$

Following Eqs. 4 and 5, we also utilized the later version of the LM test for CD by Pesaran (2015) which is better off as it is suitable for a small sample while also accounting for weak cross-sectional dependency and possible slope heterogeneity in the data set (Xu 2018).

Given the presence of the CD, we applied the CIPS panel unit root test of Pesaran (2007) which is a second-generation or augmented version of the IPS unit root test (Im et al. 2003). The examination of the level relationship was carried out with the application of the Westerlund (2007) cointegration method in line with the error correction process in Eq. 6.

6 $$\Delta {\mathrm{Y}}_{it}={\upbeta }_{i}{D}_{t}+{\uppsi }_{i}{\mathrm{Y}}_{it-1}+{\uplambda }_{i}{\mathrm{X}}_{it-1}+{\sum\nolimits }_{j=1}^{pi}{\uppsi }_{ij}{\mathrm{\Delta Y}}_{\mathrm{i},\mathrm{t}-\mathrm{j}}+{\sum\nolimits }_{j=0}^{pi}{\upgamma }_{ij}{\mathrm{\Delta X}}_{\mathrm{i},\mathrm{t}-\mathrm{j}} +{\varepsilon }_{it}$$

In Eq. 6, ascertaining the level relationship follows the outputs of the obtained panel statistics and group statistics (Pt, Pα Gt, Gα,) in line with the estimation of the error correction term (${\uppsi }_{i}$). Where the vector of parameters is denoted by ${\beta }_{t}$ while *D*_*t*_ represents the deterministic specifications which can be set at *D*_*t*_ = (0), *D*_*t*_ = (1), or *D*_*t*_ = (1, *t*), for a model with no deterministic components, with only constant component, and the one with both trend and constant, respectively. Subsequently, following the consolidated works of Koenker (2004) and Powell (2016), we applied the proposed panel quantile regression (QR) method of Koenker and Bassett (1978) to obtain the long-run coefficients while also providing a comprehensive robustness check with the Augmented Mean Group (AMG) method of Eberhardt and Bond (2009) and Eberhardt and Teal (2010). The QR representations shown in Eq. 7 relate to the nexus among variables in Eq. 1 whereby ${Q\mathrm{LnCO}2}_{it}(\tau /{\chi }_{it})$ denotes the ${\tau }^{\mathrm{th}}$ conditional quantile of the pollution levels as captured by carbon emissions among the countries. Given the determined quantile ($\tau$) for the understudied data set for country *i* at time *t*, the vector of the explanatory variables is denoted by ${\chi }_{it}$. As for the slopes of the independent variables, they are denoted by $\delta$, while ${\omega }_{it}$ represents the error term for the given vector.

7 $${Q\mathrm{LnCO}}_{2it}(\tau /{\chi }_{it})= {\delta }_{i}^{(\tau )}+{\delta }_{1}^{(\tau )}{\mathrm{LnPY}}_{it}+{\delta }_{2}^{(\tau )} {\mathrm{LnPY}}_{it}^{2}+{\delta }_{3}^{(\tau )}{\mathrm{LnFFE}}_{it}+{\delta }_{4}^{(\tau )}{\mathrm{LnRWE}}_{it}+{\delta }_{5}^{(\tau )}{\mathrm{LnATE}}_{it}+{\delta }_{6}^{(\tau )}{\mathrm{LnGZ}}_{it}+{\omega }_{it}$$

Applying both the QR methodology and AMG approaches offers certain benefits for a study like this. Firstly, the former approach is quite flexible, and it makes assessing the impacts of the explanatory variables possible on the explained variable at desired quantiles, while the latter approach offers ample insights into country-specific attributes. Secondly, both methods are preferable choices in dealing with the cross-sectional attributes of our data set (Nwaka et al. 2020).

## Results and discussions

Following the null hypothesis in Eq. 2 and given the interaction among variables in Eq. 1, the findings from Table 5 show that the CD test came out positive as the estimated significance level of the test statistics supported the rejection of the null hypothesis concerning the estimated residuals. The unit root estimates and cointegration results are in Table 6.

**Table 5:** Outputs of cross-sectional dependency test

Test approach	Breusch and Pagan (1980) LM test	Pesaran (2007) CD test	Pesaran (2015) LM test
Equation (1)	404.61a	18.52a	43.44b
Probability value	(0.0000)	(0.0000)	(0.0000)

a and b signify the statistical significance of estimates at 1% and 5% levels accordingly

**Table 6:** Unit root and cointegration outputs

Variables	*CIPS*		*IPS*			
	Intercept and trend *D*_*t*_ = *(1, t)*		Intercept and trend *D*_*t*_ = *(1, t)*			
	Levels	1st difference	Levels		1st difference	
LnCO_2_	−2.778c	−5.538a	−2.3620		−6.4322a	
LnPY	−2.596	−4.214a	−2.0264		−5.5178a	
LnPY^2^	−2.542	−4.290a	−2.2638		−3.6105a	
LnFFE	−2.436	−5.349a	−2.1396		−5.9647a	
LnRWE	−2.186	−5.084b	−2.1527		−8.4753a	
LnATE	−2.318	−5.406a	−2.5091		−8.6362a	
LnGZ	−2.505	−4.150a	−1.6790		−4.6477a	
Westerlund (2007) Cointegration						
Equation			Group		Panel	
LnCO_2_ = f(LnPY), (LnPY^2^), (LnFFE), (LnRWE), (LnATE), (LnGZ)			*Gτ*	*Gα*	*Pτ*	*Pα*
Statistics			−2.364a	−2.726a	−8.435a	−2.909a
Robust *p*-value			0.0000	0.0000	0.0000	0.0000

a signifies the statistical significance of estimate at 1% level accordingly

As seen in Table 6, there is a just basis for the rejection of the null hypothesis of no level relationship among the variables since the test statistics for the Westerlund (2007) cointegration are significant enough to make such a decision. Therefore, we explored the underlying long-run coefficients for the variables given the existence of the level relationship among them.

### Long-run estimates and causality evidence

The QR estimates in Table 7 divulge the deteriorating impacts of income level on the environmental quality among the countries as the observed coefficients are positive, significant, and also very consistent at all the conditional distribution of carbon emission levels ($\tau$ = 0.10 to $\tau$ = 0.90). This observation was also consistent with the corresponding estimates from the AMG approach that also unveils an approximate 1.7% rise in carbon emission level given a 1% growth in income level among the countries. This finding resonates with the results from some empirical studies that show that income levels can aggravate environmental degradation (Joshua and Alola 2020; Zhang et al. 2022; Adebayo et al. 2023a).

**Table 7:** QR and AMG estimations

Methods	Quantile regression (QR)									AMG
Dependent (var): LnCO_2_	$\tau$= 0.10	$\tau$= 0.20	$\tau$= 0.30	$\tau$= 0.40	$\tau$= 0.50	$\tau$= 0.60	$\tau$= 0.70	$\tau$= 0.80	$\tau$= 0.90	
LnPY	0.0802b	0.1114a	0.1372a	0.2136a	0.2900a	0.5647a	0.5994a	0.6534a	0.6558a	1.7204c
*P*-value	(0.0218)	(0.0060)	(0.0009)	(0.0000)	(0.0007)	(0.0000)	(0.0000)	(0.0000)	(0.0000)	(0.0900)
LnPY^2^	−0.0033	−0.0074c	−0.0107b	−0.019186a	−0.025307a	−0.0537a	−0.0573a	−0.0608a	−0.0615a	−0.2759c
*P*-value	(0.3573)	(0.0692)	(0.0119)	(0.0004)	(0.0026)	(0.0000)	(0.0000)	(0.0000)	(0.0000)	(0.0870)
LnFFE	0.4689a	0.5246a	0.5506a	0.4621a	0.4765a	0.7273a	0.6403a	0.4013a	0.3090a	0.9970
*P*-value	(0.0000)	(0.0001)	(0.0003)	(0.0030)	(0.0049)	(0.0000)	(0.0000)	(0.0000)	(0.0003)	(0.1590)
LnRWE	−0.1274a	−0.1189a	−0.1129a	−0.1215a	− 0.1277a	− 0.1098b	− 0.1305a	−0.1404a	−0.1892a	−0.2524a
*P*-value	(0.0000)	(0.0004)	(0.0036)	(0.0039)	(0.0050)	(0.0184)	(0.0022)	(0.0003)	(0.0000)	(0.0010)
LnATE	−0.0729a	−0.0606b	−0.0476	−0.0056	0.0612	0.3305a	0.3412a	0.3252a	0.3205a	0.0399
*P*-value	(0.0012)	(0.0316)	(0.4190)	(0.8814)	(0.3001)	(0.0000)	(0.0000)	(0.0000)	(0.0000)	(0.5450)
LnGZ	2.2566a	1.9278a	1.7185a	1.6956a	1.5362a	0.6994a	0.6491a	0.7678a	0.7225a	1.0445a
*P*-value	(0.0000)	(0.0000)	(0.0000)	(0.0000)	(0.0009)	(0.0452)	(0.0448)	(0.0091)	(0.0028)	(0.0030)
Observation	234	234	234	234	234	234	234	234	234	234
No. regressors	6	6	6	6	6	6	6	6	6	6
No. group	9	9	9	9	9	9	9	9	9	9

a, b, and c signify the statistical significance of estimates at 1%, 5%, and 10% levels accordingly

In like manner, the impacts of fossil energy consumption and globalization on the conditional distribution of carbon emission level at all quantiles ($\tau$ = 0.10 to $\tau$ = 0.90) follow the paths of the income effects, thus depicting a significant setback on the prospects of decarbonization among the countries. This result further buttresses how globalization and fossil energy consumption can be detrimental to environmental sustainability and related results have been documented in some extant studies (Erdoğan et al. 2022; Gyamfi et al. 2023; Le and Ozturk 2020) but negates findings from some other studies (Baloch et al. 2021; Shahbaz et al. 2016). This is a pointer that the integration of these countries into the globalized world has further exacerbated the production and consumption of fossil energy. Major energy-intensive sectors of their economies like transport and manufacturing sectors are vastly reliant on fossil fuels-driven technologies like automobiles and other equipment.

Furthermore, the undesirable impacts of the trio of income growth, fossil energy use, and globalization for decarbonization prospects among the countries can also be deciphered from the Dumitrescu and Hurlin (2012) causality outputs in Table 8, following the establishment of a one-way causality from income levels to emissions and a bidirectional causality from the duo of fossil energy and globalization to emission levels. This present study has focused on oil-exporting countries and it is common knowledge that oil and gas are international commodities that thrive well on the ambient of trade and economic globalization among countries. As oil production levels are sustained to meet up with revenue targets and international energy demands, domestic energy consumption is also expected to be induced over time and this is also supported by the obtained unidirectional causality from globalization to fossil energy consumption among the countries.

**Table 8:** DH panel causality test

	Zbar-Stat						
Variables	LnCO_2_	LnPY	LnFFE	LnRWE	LnATE	LnGZ	Causality scheme
LnCO_2_	_	−0.6385	4.1387a	4.1114a	3.9297a	3.2376a	$\mathrm{LnCO}2\to \mathrm{LnFFE},\mathrm{LnRWE},\mathrm{LnATE},\mathrm{LnGZ}$
LnPY	2.9065a	_	0.5707	3.1849a	0.2955	3.2729a	$\mathrm{LnPY}\to \mathrm{LnCO}2,\mathrm{LnRWE},\mathrm{LnGZ}$
LnFFE	1.9567b	2.3173b	_	2.7338b	2.7938b	1.6926	$\mathrm{LnFFE}\to \mathrm{LnCO}2,\mathrm{LnPY},\mathrm{LnRWE},\mathrm{LnATE}$
LnRWE	1.9128c	0.3644	1.3184	_	3.6393a	2.0493b	$\mathrm{LnRWE}\to \mathrm{LnCO}2,\mathrm{LnATE},\mathrm{LnGZ}$
LnATE	1.9206c	−0.2610	5.8222a	8.8534a	_	0.2356	$\mathrm{LnATE}\to \mathrm{LnCO}2,\mathrm{LnFFE},\mathrm{LnRWE}$
LnGZ	6.1939a	−0.3296	2.9665a	7.2276a	1.7532c	_	$\mathrm{LnGZ}\to \mathrm{LnCO}2,\mathrm{LnFFE},\mathrm{ LnRWE},\mathrm{LnATE}$

a, b, and c signify the statistical significance of estimates at 1%, 5%, and 10% levels accordingly

On the contrary, renewable energy use proved to be a significant tool for decarbonization prospects as its impacts were negative, significant, and highly consistent across the entire conditional distribution of carbon emission levels. The complementary results from the AMG also unwrap a significant drop of about 0.25% in CO_2_ emissions as renewables usage grows by 1%. This finding upholds some contemporary results in different studies (Anandarajah and Gambhir 2014; Pata et al. 2023; Usman et al. 2020; Erdoğan et al. 2021; Adebayo et al. 2023b). This, therefore, buttresses the urgent need for an energy transition from fossil fuels into renewables as strongly argued in many extant studies (Usman 2022a; Onifade and Alola 2022; Usman 2022b; Bekun et al. 2022). Although the proportion of the cushioning impacts of renewables is quite low compared to that of the damage created by economic growth in terms of emission, nonetheless, the results are justifiable considering that many of these countries are still at their emerging economic status with more attention been focused on income expansion even if such expansion hinges on a deteriorating environment. As for alternative energy use, the evidence is very mixed across quantile distribution. While alternative energy use significantly reduces emission levels at lower quantiles ($\tau$ = 0.10 and $\tau$ = 0.20), however, when considering the intermediate quantiles ($\tau$ = 0.40 to $\tau$ = 0.50), the impacts were not significant until the upper quantiles.

The AMG results lend credence to this inconsistency as the estimate was likewise insignificant for this variable. This outcome is not a surprise considering that the proportion of alternative energy in total energy is abysmally low among the countries. Nuclear energy consumption has little or no attention in most of the understudied countries to create any overall significant effect on environmental sustainability through carbon emission levels. This is not the case in some developed economies like the USA, where significant economic and environmental benefits of nuclear energy have been reported in the literature (Ozturk 2017; Kartal et al. 2023; Duran et al. 2022). Furthermore, a look at the country-specific analysis in Table 9 also provides more insights in this regard. Additionally, a clearer picture of the extent of the validity of the EKC for an individual country is detailed in the country-specific analysis in “Country-specific estimations,” while an overall graphical scheme of results is depicted in Appendix Fig. 3. Lastly, the QR technique passes the diagnostic test of slope equality that was conducted. The chi-square statistic for the Wald test was 233.58 with a *P*-value of 0.0000, thus supporting the rejection of the assumption of slope homogeneity according to the null hypothesis. As such, there is a significant variation in the obtained slope parameters across quantile levels.

**Table 9:** AMG outputs for country-specific estimations

Explained variable	Explanatory variables					
(LnCO_2_)	LnPY	LnPY^2^	LnFFE	LnRWE	LnATE	LnGZ
Countries	Coefficients					
Egypt	2.5072c	−0.3762c	2.8481	−0.3609	−0.0847	0.9029c
Algeria	−3.2101c	0.4569c	−51.0644	−0.1517b	0.0487	−0.0211
Sudan	2.0684c	−0.3903c	−0.0611	−3.1058a	−0.1324b	0.9631a
Tunisia	0.3518	−0.0383	−1.9084	−0.2460	0.0489a	1.4263a
Nigeria	3.2429b	−0.5350b	1.8892a	−0.2669	−0.07032	2.5587a
Angola	0.6988	−0.0645	−0.3790	0.3369	−0.0615	1.4727
Congo Rep	−7.6607c	1.1975c	1.6434a	3.8768a	0.7522b	2.2400
Gabon	7.1361b	−0.9264b	0.2203	−0.1732	0.2437	−0.1160
South Africa	1.6729	−0.2264	3.7046b	−0.4680b	0.2166c	0.0468

a, b, and c signify the statistical significance of estimates at 1%, 5%, and 10% levels accordingly

### Country-specific estimations

In Table 9, fossil energy consumption specifically worsens environmental degradation levels as it creates positive impacts on emission levels among some of the countries including Egypt, Gabon, Nigeria, Congo Republic, and South Africa. The magnitude of the obtained pollution inducement by fossil fuel usage was highest in the case of South Africa, followed by Egypt, Nigeria, and Congo respectively. The positive impacts were however not significant in the specific cases of Egypt among the others. In this context, the specific findings for Nigeria and South Africa are not surprising given that the duo is the top carbon emitter on the continent courtesy of the oil and gas (for Nigeria) and coal energy consumption (for South Africa).

On the other hand, renewable energy use has negative impacts on carbon emissions in many of these countries except for the cases of Angola and the Congo Republic and the pollution abatement impacts are significant in the specific case of Algeria, Sudan, and South Africa. On the other hand, while there were negative impacts of alternative energy use in Egypt, Nigeria, Sudan, and Angola, these impacts were only significant in the specific case of Sudan. It is worth noting that the proportion of alternative energy in total energy consumption has specifically steadily grown in Sudan in relative comparison to others. For instance, according to the World Bank database, among the North African countries in the study, alternative energy use accounted for approximately 3.5% of the total energy use in Sudan between 2010 and 2015, while it was just 0.04%, 0.60%, and 1.46% in Algeria, Tunisia, and Egypt. Thus, even though nuclear energy consumption has little or no attention in most of the understudied countries to create any overall significant effect on environmental sustainability through carbon emission level, the country-specific analysis has further revealed more insight into this particular variable.

Furthermore, globalization significantly hampers the environment via emission inducement with the highest magnitudes in the specific case of Nigeria, Tunisia, Sudan, and Egypt. Finally, the country-specific analysis gives credence to the validity of the EKC hypothesis in Egypt, Sudan, Nigeria, and Gabon thus supporting the findings of Mahmood et al. (2019). For South Africa, Angola, and Tunisia, the income coefficients follow the expected directions but were not statistically significant to uphold the EKC, thus contradicting the results by Shahbaz et al. (2014). For Congo and Algeria, the U-shape hypothesis was confirmed.

## Conclusion and policy directions

The impacts of the energy mix on the quality of the environment were examined among oil-producing nations in Africa towards addressing the global desire for decarbonization while also throwing light on the economic aspects of such prospects. The impact analysis is hinged on quantile regression (QR) and Augmented Mean Group (AMG) estimators in deciphering the effects of the energy indicators on carbon emission levels across the countries between 1990 and 2015. The study utilized data from oil-producing African nations including Algeria, Nigeria, Angola, Egypt, Tunisia, Gabon, Congo Republic, and Sudan. In addition, the analysis was also extended to cover the unique case of South Africa being the leading carbon emitter on the continent with vast fossil resources in terms of coals.

The empirical analysis was done via the adopted methodologies while also providing additional details on the country-specific setting. According to the empirical results, among the countries, only renewable energy sources support decarbonization of the environment by 0.25% with respect to a percentage rise in their usage. Moreover, the consequences of the trio of fossil fuel consumption, income growth, and globalization are diametrically opposed to achieving decarbonization as the rise in their usage significantly acts as pollutant-inducing tools. These undesirable impacts of the trio of income growth, fossil energy use, and globalization for decarbonization prospects among the countries were further deciphered by the causality evidence. Additionally, the study establishes the EKC hypothesis even with the specific case of the individual countries.

### Policy insight

Decarbonization has desirable outcomes as it is a critical step towards addressing environmental problems vis-a-vis averting possible damages of climate change which are even expected to be more catastrophic for less developed African economies. Therefore, given the aforementioned findings, we recommend concerted efforts for more investment in renewable technologies from both the public and private sectors, especially in sources like solar and wind energy generation. These sources are not only renewable but also feasible and sustainable alternative energy sources for the countries in the study and by extension to other countries on the continent given Africa’s strategic geographical advantages. For instance, it has been noted that Africa has about 10-TW capacity of solar energy potential with other exploitable energy capacities for sources like wind, hydro, and even geothermal estimated to be about 110 GW, 350 GW, and 15 GW, respectively (United Nations Environmental Program 2017). Sadly, this huge potential is still largely untapped and as such offers more prospects for the continent. Some oil-producing Northern African countries in the study like Algeria, Egypt, and Tunisia have higher advantages in the development of both solar and wind energy.

Furthermore, the understudied countries, and most especially those that are highly notable with substantial oil and gas revenues like Nigeria, Angola, and Algeria, should also take advantage of the oil proceeds to kick start necessary green energy projects. The levelized cost (LC) of investment for such sustainable projects can be offset with necessary support from the oil industry. We understand the initial cost could be high, but then, that is where the advantage of the oil revenues is expected to come in. The nation can strategically set up special green energy investment funds and such funds should cover a long-term design to harness the benefits of windfall revenues from oil and gas for sustainable energy transition programs towards a post-oil era. Also, given the alarming cases of corruption in public spheres on the continent as well documented in the literature (Hope 2020; Sassi and Ali 2017), policy measures and legislation must be put in place to strengthen relevant institutions towards ensuring prudent management of green energy investment funds.

Finally, authorities and policymakers of the understudied countries can harness globalization for their environmental benefits. In this regard, the countries can make use of their trade policies to foster decarbonization prospects. For instance, carefully designed trade policies that support a rise in the adoption and utilization of environmentally beneficial technologies can be implemented to further reduce the dependence on fossil energy utilization. This is very important for all the understudied countries and more especially for the specific cases of Egypt, Nigeria, Congo, and South Africa where the magnitude of the obtained pollution inducement by fossil fuel usage is substantially high. Encouraging green technological transfers via the instrumentality of globalization would help to boost the environmental well-being of these countries in the long term.

## Figures and Tables

**Fig. 3:**
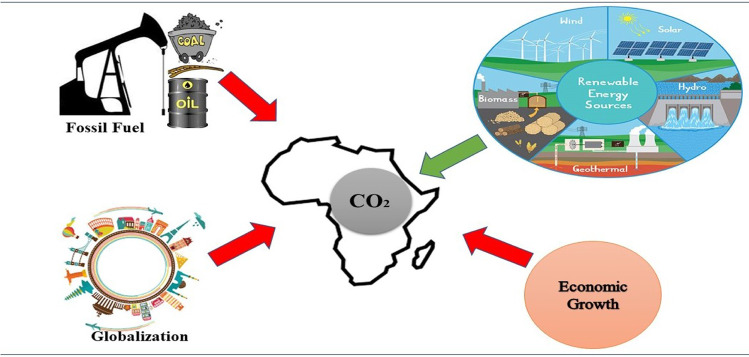
Overall graphical scheme of results. Green and red arrows indicate negative and positive impacts respectively

## Data Availability

Not applicable

## Appendix

Figure 3

## Abbreviations

EKC: Environmental Kuznets curve
EU: European Union
UN: The United Nations
GHG: Greenhouse gas
WDI: World Development Indicators
CO_2_: Carbon dioxide
UNEP: The United Nations Environment Program
SDGs: The United Nations sustainable development goals
CH4: Methane
COP27: Conference of the Parties of the UNFCCC
OECD: Organization for Economic Cooperation and Development
R&D: Research and development
OLS: Ordinary least squares
ECT: Error correction term
EGR: Emission gas report
ARDL: Autoregressive distributed lag
FMOLS: Fully modified ordinary least square
DOLS: Dynamic ordinary least square
BRICS: Brazil, Russia, India, China, and South Africa
UNFCCC: The United Nations Framework Convention on Climate Change
UK: United Kingdom
N2O: Nitrous oxide
PMG: Pooled Mean Group
mb/d: Million barrels per day
OPEC: The Organization of the Petroleum Exporting
IPCC: The Intergovernmental Panel on Climate Change
CIS: Commonwealth of Independent States
CD: Cross-sectional dependency
GDP: Gross domestic product
BP: British Petroleum
AMG: Augmented Mean Group
QR: Quantile regression
